# Identifying and managing apathy in people with dementia living in nursing homes: a qualitative study

**DOI:** 10.1186/s12877-023-04422-y

**Published:** 2023-11-09

**Authors:** Johanna M. H. Nijsten, Martin Smalbrugge, Annette O. A. Plouvier, Raymond T. C. M. Koopmans, Ruslan Leontjevas, Debby L. Gerritsen

**Affiliations:** 1 Radboud University, Nijmegen, The Netherlands; 2 Department of Primary and Community Care, Research Institute for Medical Innovation, Radboud University Medical Centre, Nijmegen, The Netherlands; 3 Radboud Alzheimer Centre, Nijmegen, The Netherlands; 4 Archipel, Landrijt Expertise Centre for Specialized Care, Eindhoven, The Netherlands; 5 Department of Medicine for Older People, UMC, Location Vrije Universiteit Amsterdam, Amsterdam, The Netherlands; 6 Aging & Later Life, Amsterdam Public Health Research Institute, Amsterdam, The Netherlands; 7 Waalboog, Joachim and Anna, Centre for Specialized Geriatric Care, Nijmegen, The Netherlands; 8 Faculty of Psychology, Open University of the Netherlands, Heerlen, The Netherlands

**Keywords:** *Apathy*, *Caregivers*, *Dementia*, *Nursing homes*, *Well-being*, *Qualitative research*

## Abstract

**Background:**

Although apathy is common in people with dementia and has profound negative effects, it is rarely diagnosed nor specifically treated in nursing homes. The aim of this study is to explore experiences in identifying and managing apathy from the perspectives of people with dementia and apathy (PwA), family caregivers (FCs) and professional caregivers (PCs).

**Methods:**

Descriptive qualitative study with purposive sampling, comprising eleven semi-structured in-depth interviews with PwA, FCs or PCs and focus groups with twelve PCs in Dutch nursing homes. Seventeen additional in-depth interviews with caregivers were held, after signals of increasing apathy during the first Covid-19 lockdown. Using an inductive approach, data was analysed thematically to explore the experiences in identifying and managing apathy from the perspective of different stakeholders.

**Results:**

Three themes were identified: 1) the challenge to appraise signals, 2) the perceived impact on well-being, 3) applied strategies to manage apathy. Although participants described apathy in line with diagnostic criteria, they were unfamiliar with the term apathy and had difficulties in appraising signals of apathy. Also, the perceived impact of apathy varied per stakeholder. PwA had difficulties reflecting on their internal state. FCs and PCs experienced apathy as challenging when it reduced the well-being of PwA or when they themselves experienced ambiguity, frustration, insecurity, disappointment or turning away. Dealing with apathy required applying specific strategies that included stimulating meaningful contact, adjusting one’s expectations, and appreciating little successes.

**Conclusions:**

When addressing apathy in nursing homes, it is important to consider that a) all stakeholders experience that appraising signals of apathy is challenging; b) apathy negatively influences the well-being of people with dementia and especially their FCs and PCs; and c) FCs and PCs can successfully, albeit temporarily, manage apathy by using specific strategies.

**Supplementary Information:**

The online version contains supplementary material available at 10.1186/s12877-023-04422-y.

## Background

Apathy comprises cognitive, affective and behavioural symptoms and is described as diminished interest, emotional expression/responsiveness or initiative, which causes significant functional impairment and reduced qualitative participation in daily living, social contact or activities [1, 2]. While apathy, depression and cognitive decline often co-occur, they are regarded as separate entities [3, 4]. Reduced interest, initiative and decreased motivation are overlapping symptoms in apathy and depression that sometimes makes the differentiation between them difficult. However, apathy and depression are considered distinct clinical syndromes. Sadness and feelings of helplessness, hopelessness and worthlessness are typical for depression and not for apathy [1, 5]. In addition to the neural and neurobiological mechanisms (e.g., frontal-striatal circuits) associated with neurocognitive disorders, other factors can contribute to apathy. These include individual factors like neuropsychiatric symptoms (NPS), caregiver factors like stress and burden, communication issues, unrealistic expectations or a lack of knowledge and environmental factors such as the presence of activities and structure, and over- or under-stimulation [6–8].

Apathy is common in people with neurological and neurodegenerative diseases, affecting approximately 50% of nursing home (NH) residents with and without dementia [9–11]. In people with dementia, apathy is the most common neuropsychiatric symptom and its prevalence is similar throughout different dementia stages (54% in mild, 59% in moderate and 43% in severe dementia) or subtypes (60% in Alzheimer Disease, 60% in Vascular Dementia) [9, 12]. Furthermore, apathy is known to be persistent and associated with functional and cognitive decline, and apathy is a risk factor for increased mortality [10, 13–15]. Nevertheless, despite its profound negative effects, and in contrast to other NPS, apathy in people living in NHs is seldomly explicitly diagnosed nor specifically treated.

Despite growing scientific interest, the impact of apathy on well-being for people with dementia in NHs and their family caregivers (FCs) and professional caregivers (PCs) remains unclear. In people with dementia living at home, apathy has been associated with an increased reliance and burden on caregivers, as well as earlier admission to a NH [16–18]. Previous research has found that from the proxy perspective apathy is negatively associated with health-related quality of life in young as well as late onset dementia [19, 20]. Furthermore, the literature on the effect of apathy on self-reported quality of life in people with dementia living in NHs is inconsistent [19, 21]. Some studies suggest reflecting on apathy can be very difficult for people with dementia due to lack of insight and awareness, resulting in apathy having no effect on self-reported quality of life [19, 21], while other studies suggest that apathy can be seen as a coping strategy of persons with dementia to protect themselves from confrontation with failure, and disappointment [22, 23]. Moreover, although FCs from community-dwelling people with dementia struggle to cope with apathy of their loved-ones [24], in NHs apathy is seldom reported as a problem by persons with dementia themselves or PCs. Behaviour like agitation, aggression or depression interfere with work routines and demand attention and therefore are likely to trigger action from PCs. In contrast, apathy is characterized by diminished or absent behaviour and emotion, that does not trigger action easily and is therefore seldom reported as a problem by PCs [25–28].

There is currently no distinct pharmacological or psycho-social treatment for apathy [29, 30]. Some psycho-social interventions are promising when provided in multidisciplinary [30] like adapted physical activity[31], therapeutic activities [32, 33] and music therapy [34]. Psycho-social interventions are the first choice when managing apathy [35, 36]. Indeed, although apathy is very prevalent in NHs [11], has distinct negative outcomes and is commonly part of the assessment of neuropsychiatric symptoms in dementia, diagnosing and targeting apathy by specific treatment is uncommon in daily nursing home practice [30, 37, 38].

In this study, we therefore explore the experiences of persons with apathy (PwA), their FCs and PCs in identifying and managing apathy in NHs.

## Methods

### Study design

In the Shared Action for Breaking through Apathy project (SABA), an intervention to identify and manage apathy in NHs was co-created together with PwA, FCs, and PCs. In this paper, we report on the first step towards creating the intervention. To study people’s subjective attitudes, opinions, beliefs and reflections we used a generic qualitative research design with an inductive and descriptive approach [39]. Founded on main principles in qualitative research, the generic qualitative design uses methods adopted from established qualitative approaches, such as data triangulation and the constant comparative method [40, 41]. Based on purpose sampling, we held face-to-face semi-structured interviews with PwA, their FC(s) and different PCs to get broad insight into participants’ experiences. The FCs (all legal representatives) of PwA were approached after multidisciplinary screening indicated that their loved one had apathy. They were provided with written and verbal information by the local psychologist and asked for permission to participate. Thereafter, an interview with the PwA and their FC(s) was scheduled by the researcher (HN). The PwA was interviewed in the own apartment with their FCs present. Before the interview the PwA and their FCs were informed on the study and were able to ask questions.

PCs were approached by the local coordinator of each participating organization to participate. They were given verbal and written information on the study. Thereafter, they were approached for an interview with researcher HN. Subsequently, in the iterative process, we held focus groups with PCs, to further explore the topics addressed in the interviews and explore multidisciplinary viewpoints. Before each interview and focus group discussion, verbal information was given and participants were able to ask questions. During restrictive measures (visitor ban) in NHs due to the Covid-19 pandemic, apathy seemed to be more profound in people with dementia [42]. As this may have broadened or deepened their experiences with apathy, we held additional interviews with a FC and with PCs who had specifically mentioned effects on apathy in an online survey on behavioural changes during lock-down [43]. The PCs had volunteered to be approached for an interview. They were given written information. Moreover, verbal information was provided before the interview and they were able to ask questions on forehand through mail and at the start of the interview. This study was described using the Consolidated Criteria for Reporting Qualitative Research (COREQ) checklist [44] (see Additional file 1).

### Setting and participants

Two Dutch care organizations of the University Knowledge Network for Older Adult Care Nijmegen participated in this project. Residents classified as having Alzheimer’s dementia, vascular dementia and dementia not otherwise specified were included and screened for apathy.

A physician and/or psychologist evaluated all residents in the participating dementia special care units and selected which of them showed apathy symptoms. Those selected were screened with the shortened Apathy Evaluation Scale (AES-10) [45] by a nurse and psychologist familiar with the resident. The ten items of this validated observational scale vary from 1 (not at all characteristic) to 4 (very characteristic), whereby a higher sum score reflects more apathy symptoms (range 10–40). If the AES-10 score indicated apathy (> 21) [45], the physician and psychologist ruled out those with apathy due to untreated depressive disorder, acute illness or medication, or apathy representing a resident’s character rather than a symptom. As our previous research showed that apathy can best be considered as a dimensional construct [10] the AES-10 was used to assess the severity of apathy, rather than the often used subscale of the Neuropsychiatry Inventory that assesses apathy categorical and has moderate validity [46, 47]. Additionally, the physician registered the type of dementia based on the medical file and the severity of dementia using the validated Global Deterioration Scale (GDS) [48]. The GDS describes seven stages of cognitive decline in primary degenerative dementia from mild cognitive impairment (stage 1) to severe dementia (stage 7). Of the residents who met the inclusion criteria, a nurse and psychologist familiar to the resident estimated which of them would be able to participate in an interview and communicate about their experiences.

A purposive sampling process was used to recruit a representative sample of PCs involved in daily care. They were invited for an individual interview and/or focus group. The sample comprised of nurse assistants, nurses, specialized nurses, psychologists, physicians, and activity coordinators (at least one per participating organisation) with variation in age, sex, cultural background and educational level.

### Data collection

Those PwA and their FC were then approached for participation by the same nurse or psychologist. PwA and their family member were interviewed together by the interviewer (HN) in the resident’s own room at the unit of residence in September 2019.

Between September and December 2019, PCs were interviewed separately by the same interviewer (HN) on site. Moreover, focus groups with PCs were held at location and moderated by two moderators (AB + HN; HN + AP). All interviews and focus groups were audio recorded with permission of the participants. To increase the trustworthiness of results, ideally the findings should be confirmed with participants [49]. However, none of the interviewees wanted a written member check, but a verbal summary and member check was performed at the end of each interview.

The guides for the interviews and focus groups were compiled by the research team (HN, AP, DG; see Additional file 3). The topics discussed were the experiences of the interviewees in recognizing and dealing with apathy and whether and how apathy was burdensome to them. Open responses were encouraged in all interviews and focus groups. Input from the interviews was used in two multidisciplinary focus groups with PCs to further explore and discuss possible different viewpoints for generating a wider range of ideas and perspectives. The data collection proceeded until constant comparison analysis revealed that information saturation was achieved, which was defined as no new codes generated from the interviews or focus groups.

The additional interviews were held between June and September 2020 with a FC and PCs. The initial topic guide was adapted for these interviews to explore experiences in recognizing and dealing with apathy in the specific context of the lock-down in which apathy appeared to become more prevalent [43] (see additional file 3). Due to ongoing restrictions, these interviews (HN, AP) were held using digital connection (Zoom, MS Teams) and audio-recorded.

The research team was a multidisciplinary team consisting of members with a medical (MS, RK, AP) or psychological background (HN, RL, DG) all of whom have experience in older adult care and qualitative research.

### Data analysis

Data were analysed concurrently with the data collection. After transcribing the interviews and focus groups verbatim, the data were anonymized and analysed using Atlas.ti (version 8.4.20) using inductive thematic analysis [41, 50]. Two researchers (HN, AP) independently derived codes from the data and discussed them until they reached consensus. The codes were then, independently by each researcher, grouped into higher-order categories based on meaning or content. To enhance the process of achieving consensus and analytic rigour, the researchers (HN, AP) engaged in a reiterant process of discussing areas of agreement and disagreements. The final themes were discussed with the research team (DG, MS, RK, RL) to reach consensus on the themes that characterize the different stakeholders’ experiences in identifying and managing apathy.

### Ethical considerations

The study was conducted according to the guidelines of the declaration of Helsinki and approved by The Medical Ethics Review Committee (CMO Arnhem-Nijmegen) region (File number 2019–5539) and the local ethical committees of the participating organizations. All participants were informed and gave written informed consent before participation. For the additional interviews during the COVID-19 pandemic, informed consent was given verbally by the participants and audio recorded. During the recruitment of participants for the study in this paper, it became clear the PwA were unable to give informed consent due to cognitive and communication issues. With permission of CMO Arnhem-Nijmegen (file number 2019–5539), we than adjusted the inclusion procedure for PwA in the second step of SABA (outside the scope of this paper). In this step (the development and feasibility of an intervention) informed consent was provided by the legal representatives of PwA. Further details on this step are presented elsewhere [51].

## Results

### Participants’ characteristics

After screening all 117 residents of participating units, 34 residents were suspected of apathy and therefore evaluated multidisciplinary. Of the nine residents with an apathy-indicating AES score, six were able to communicate about their apathy. They were invited with their FCs to participate in an interview. Two of them gave informed consent. Reasons for not participating were no interest (*N* = 2), deceased (*N* = 1) and unknown (*N* = 1). The two residents with dementia who were able to communicate about their apathy and willing to participate were interviewed together with one or two FC(s). The residents had moderately severe dementia according to the GDS (stage 5 and 6, respectively) and AES scores of 29 and 30, respectively. Six PCs were interviewed individually (two of each specific profession) and two focus groups were held with five and seven PCs, respectively. The PCs were nurse assistants, nurses, specialized nurses, psychologists, physicians, and activity coordinators (at least one per participating organisation) with variation in age, sex, cultural background and educational level. One FC and sixteen PCs participated in the additional interviews (see Table 1 for participants’ details). The interviews lasted between 39 and 67 min and the focus group discussions lasted 90 min.

**Table 1:** Demographic characteristics of people with dementia and apathy, family caregivers and professional caregivers

	**Participated in interview *n* {% of interviewees}**	**Participated in focus groups *n* {% of focus group participants}**	**Participated in additional interview *n {*% of interviewees}**	**Age range**	**Sex *n {*% female}**	**Educational level {n}**
*People with apathy*	2 {18,2}	-	-	84–93	2 {100,0}	Low {2}
*Family caregivers*	3 {27,3}	-	1 {5,9} [1**]	60–64	2 {66,7}	Middle {1}
						High {2}
*Professional caregivers*						
Care / nurse assistant	-	3 {25,0}	-	30–57	3 {100,0}	Low {1}
						Middle {2}
Nurse {in training}	2 {18,2}	2 {16,7} [1*]	5 {29,4} [1*; 1**]	33–65	7{77,6}	Middle {8}
						High {1}
Specialist nurse {in training}	-	3 {25,0}	-	20–47	2 {66,7}	High{2}
Activity coordinator	-	-	3 {17,6}	27–37	3 {100,0}	Middle {3}
Psychologist	2 {18,2}	2 {16,7}	6 {35,2} [1**]	24–63	9 {77,8}	High {9}
Physician	2 {18,2}	2 {16,7} [1*]	2 {11,8} [1***]	32–54	3 {100,0}	High {3}
**Total**	11 {100}	12 {100}	17 {100}			

[n*] = participated in one interview and a focus group; [n**] = participated in two interviews; [n***] = participated in two interviews and a focus group

### Qualitative findings

#### Themes and subthemes

Based on the views of PwA, FCs and PCs, we identified three central themes regarding apathy in NHs that help to understand how the identification and management of apathy is experienced (1) the challenge to appraise signals, (2) the perceived impact on well-being, and (3) applied strategies to manage apathy. Below, each theme is discussed and illustrated with meaningful quotes from the interviews and focus groups. See Table 2 for an overview of themes and subthemes and Additional file 2 for additional quotes.

**Table 2:** Overview of themes and subthemes

Themes	Subthemes
The challenge to appraise signals	Perceiving loss of emotions and behaviour
	The importance of knowing the context
	Apathy as part of dementia
The perceived impact on well-being	Perceived impact of apathy on well-being of a PwA
	Perceived impact of apathy on the well-being of FCs and PCs
Applied strategies to manage apathy	Stimulating meaningful contact
	Adjusting expectations
	Appreciating little successes

*PwA* Person with apathy and dementia, *FC* Family caregiver, *PC* Professional caregiver

### Theme: “The challenge to appraise signals”

One of the themes that was identified from the analysis was the challenge of appraising signals of apathy adequately and as being relevant. This theme included perceiving a loss of emotions and behaviour, the importance of knowing the context and apathy as an undeniable part of dementia.

#### Perceiving loss of emotions and behaviour

Stakeholders mostly described apathy as a decrease or absence of emotions, behaviour or engagement. This absence of signals makes it difficult for them to see which emotions, behaviour or engagement are relevant because it requires seeing what’s not or no longer there. PwA described a ‘*loss of initiative’,* ‘*indifference’* or stated ‘*I don’t feel like it*’, but they were unfamiliar with the term ‘apathy’ when asked specifically. Some PCs mentioned being familiar with the term apathy, whereas others were not. Several FCs and PCs described apathy as a decrease in emotional reaction, a lack of initiating behaviour or social engagement as well as a person’s body language ‘*just sitting*’.

*‘At a given moment our mum sat on a chair and just stayed seated.’ (FC,001)*

*‘I did nothing, I didn’t do anything anymore.’ (PwA,001)*

*‘Sometimes they [people with apathy] are slightly aware of the environment, they are very turned inward, sometimes I see them looking around but not really interacting. You don’t see them engage in an activity. They just sit there. They are people about whom I always think: if it got dark, they would just remain in the dark because they wouldn’t take the initiative to get up and switch on the light.’ (PC,004)*

When asked, people with dementia mentioned that they did not know why they did not initiate action or dropped out from activities, and one woman with apathy mentioned that she “*just didn’t think about it*”.

According to FCs and PCs, PwA frequently dropped out from activities offered to them or prematurely withdrew from social interaction. They mainly ascribed this to the inability to express and fulfil one’s actual needs as a result of apathy, rather than being unwilling to interact or engage.

*‘I used to do needlework like this, knitting the most beautiful sweaters, and crocheting, and now I don’t do anything.’ (PwA,002)*

*‘And apathy in terms of, like, not being able to get things started, it’s not always that the person is okay with it or doesn’t feel like it or doesn’t want to, but that’s just a part of it: they simply can’t do it.’ (PC,001)*

*The importance of knowing the context.* Apathy was described by all stakeholders as being recognizable by a change compared with the way in which a person with dementia was before. PCs mentioned the importance of knowing the personal life history and personality of a PwA in order to compare character traits from the past to observed current behaviour. Moreover, they expressed using observation and conversation to check apathy symptoms after ruling out other probable causes for the observed behaviour, such as depression or side effects of medication.

*‘It’s often when things change that it’s [apathy] more noticeable and then you will discuss it. Yes, it’s only really noticeable if the change is significant.’ (PC, 007)*

PCs mentioned that a sudden change and reduction in environmental stimuli due to the COVID-19 lockdown made apathy more apparent and visible for them, and that this was mostly reversed when the lockdown ended and activities and social contact were re-established. A PC described that people were more in their own world and less easy to stimulate because normal activities and social contact had disappeared “*as if the daily wheel of rhythm’ stopped and had to be restarted*” *(PC 004).*

*‘What struck me most is that you really can see that when a lot is omitted, if- due to lockdown – there are no family, no volunteers, no activities and a lot of things have to be done by protocol, then you see that apathetic behaviour increases a lot. Yes, you just notice that, also in people you wouldn’t expect.’ (PC,002)*

*Apathy as an undeniable part of dementia.* Both caregiver groups mentioned that they see dementia as a probable and natural cause of apathy, making it difficult to distinguish or recognize apathy as a separate entity that needs attention. However, at the same time they realize that apathy should be addressed if it negatively affects the person with dementia or those around them. PwA did not mention this subtheme.

*‘But in cases of people with severe dementia, don’t they act because they no longer can, or because they're hindered by their apathy, and is there any difference between these? [….] Or do we still call that apathy and just no longer think that’s something bad?’ (PC,003)*

### Theme: “The perceived impact on well-being”

The second theme that emerged from the interviews regarded the perceived impact of apathy on well-being. PwA only referred to their own well-being, while FCs and PCs reflected on the well-being of the PwA as appraised from their proxy perspective, as well as their own well-being.

#### Perceived impact of apathy on the well-being of the person with dementia

The persons with dementia described the impact of apathy on well-being as a loss of interest and feeling indifferent towards activities. They agreed when FCs or the interviewer mentioned examples, but did not mention examples or express a burden themselves.

*‘If we talk about it like this, about apathy, does it bother you like it is now? (Interviewer) Sometimes it does, sometimes it doesn’t. It probably depends on, how shall I say, it depends on how I’m feeling’. (PwA,002) And what influences that? (Interviewer)I don’t know. If I lay down or sit a lot…Yes, then I also nod off.’ (PwA,002)*

FCs and PCs mentioned that it was difficult for them to evaluate the well-being of a person with dementia and apathy as they often did not express emotions or burden.

*‘It looks like residents don’t suffer from apathy because they lack the insight into what’s the matter with them, so they can’t express themselves like: ’Well I’m here all day long not doing anything at all’. They can’t really say. So, it more or less depends on the observations that we or I do.’ (PC,002)*

*‘I think it differs whether you see somebody with apathy who at the same time looks miserable or makes an unhappy impression, or if somebody is without initiative but at the same time looks relaxed’ (PC,006)*

On the other hand, caregivers emphasized that they imagine it must be disturbing to have apathy and not be able to take initiative or express oneself. FCs and PCs felt that people with apathy must experience apathy as lack of meaning in life.

*‘Yes, burden is a big term, I do think it bothers them, but they can’t really express themselves. I can’t imagine otherwise than it must bother you if you don’t initiate anything. That – to me – seems very disturbing, but I think they can’t express that*.’ *(PC,005)*

*‘Well, if there’s no meaning in life at all, and people just shut down from everything and everybody, then there’s nothing left at all. And every human being deserves to feel that they are allowed to be there until the very end.’ (PC,002)*

Both caregiver groups stated that apathy became more challenging and triggered them to react when they estimated that apathy reduced the well-being of the person with dementia or led to further deterioration. Especially when a sad mood was noted in combination with apathy, this was considered burdensome for the person with dementia.

*‘Well, you must have the impression that there is some kind of, yes, some kind of suffering by someone, by the resident, by the caregiver or by the family. So somewhere there has to be some kind of, kind of burden, yes, before any real action is taken.’ (PC,007)*

*‘I worry because I think that it [apathy] is just something very negative for a resident. Those people who suffer from apathy, they don't experience enough stimuli because they don't seek it themselves. So, what you get is that someone only deteriorates further. So, you feel like: I need to activate someone. That's very much the feeling I get from someone, like, go, go and do something!’ (PC,001)*

#### Impact of apathy on the well-being of the FCs and PCs

Both caregiver groups described that visiting and interacting with or taking care of a person with dementia and apathy can be challenging and lead to frustration, disappointment, insecurity or turning away.

*‘Well, you can keep asking, there is no reaction. If I sit [with her] for an hour and I ask different things, then no, you won’t get a response, so you really do not know: “am I doing this right, half right or am I not doing it right at all?”’ (FC,003)*

*‘I think it is difficult because, well, sometimes you want something [in contact] and if there is nothing, it is a kind of frustration you have to manage and be patient, and sometimes it just won’t work*.’ *(PC,007)*

FCs felt that apathy might not be very challenging for PCs as the PwA would not demand a lot of attention. PCs mentioned apathy to be some kind of challenging behaviour, albeit not a very burdensome one.

*‘Our mom is very quiet, so maybe she’ll be more easily ignored for certain things, because the others require a lot more attention.’ (FC,002)*

*‘I think it's some kind of challenging behaviour, but it's not a behaviour that causes a challenge for us.’ (PC,005)*

FCs and PCs emphasized that it was very rewarding for them when they were able to overcome apathy in a person with dementia. When they establish some kind of reaction, even if short-lived, this positively reflected on their own feelings.

*‘Yes, how nice it is when you see somebody with apathy smile for whatever reason, because of something you said or just a reaction or becoming happy, even if it only lasts for a short while. That, I find beautiful*.’ *(PC,008)*

### Theme: “Applied strategies to manage apathy”

The third theme referred to the skills and capabilities of FCs and PCs to apply strategies to manage apathy. FCs and PCs used different skills and capabilities to manage apathy in a person with dementia that were sometimes used consciously and sometimes more unconsciously.

#### Stimulating meaningful contact

FCs and PCs described that they stimulated the person with dementia into some kind of action or response to overcome the lack of meaningful interaction they experienced as a result of the person’s apathy. This was described as the motivation to keep trying to stimulate the person with dementia into some kind of action or positive response.

*Because these things [person-centred activities], if it works, that’s what secretly you’re always looking for: that you can give somebody a good time and make them feel good.’ (PC,006)*

FCs and PCs emphasized the importance of person-centred activities that refer to former and familiar routines, interests or hobbies. By supporting communication in a non-verbal manner, meaningful contact was realized more easily, especially with people with more advanced dementia.

*Well, it depends on the [dementia] stage of a person. We had a resident with quite advanced dementia, but when he heard his name and saw his wife in a video call, then he really revived. I saw a twinkle in his eyes and I noticed the recognition was there. And that family was very good in communicating in a non-verbal way: waving, blowing kisses, blinking, showing the dog. One could see the man really perk up. That was very nice.’ (PC,002)*

FCs and PCs talked about how people with apathy were stimulated to engage in activities by simply taking them to an activity or starting a specific activity rather than asking if they wanted to participate. The interviewed people with apathy agreed with this statement. This strategy was based on experiences that people with apathy enjoyed an activity once they started participating. Moreover, PCs mentioned examples in which they started the activity ‘*like starting the engine*’ and once having started, the PwA could continue by him-/herself for a short while.

*‘So, when it [an activity] is unfamiliar, it’s hard to participate? (Interviewer) ‘Yes, I’m just not in the mood for it. But if somebody asked: 'Would you put a needle into this for me [for sowing]?” I would do so.’ (PwA,002)*

*‘There are people of whom you know that – once you get them involved in an activity – they really enjoy it, but they can’t take the initiative themselves somehow. It’s a pity when it [inviting to participate in an activity] does not work, because I know, afterwards they would have enjoyed it, they would have had such a pleasant afternoon, or hour. So yeah, you really want it to work.’ (PC,006)*

#### Adjusting expectations

Another strategy in dealing with apathy mentioned by FCs and PCs was adapting their own expectations to be more realistic and, the capability to change their own behaviour when taking care of or visiting a person with dementia and apathy.

*‘But then I realized it’s the way it is. She no longer is able to, willing to [participate]. She won’t do it herself, so I need to change myself.’ (FC,001)*

*‘You’ll always aim high, yet when you are dealing with someone with apathy, you shouldn’t aim too high, start low.’(PC,009)*

The importance of keeping a balance between stimulating and letting the PwA be was emphasized by both caregiver groups. This also meant that a caregiver sometimes needed to accept apathy in a person with dementia temporarily. One PwA described that simply sitting with other people without actually participating in conversation was pleasant enough for her.

*‘If I know I have done everything, tried everything, then automatically I come to realize: ‘It is what it is’. This does not mean that I accept it [apathy] or won’t put effort into it, but I can leave it be for a while and then start over again later on.’ (PC,002)*

### Appreciating little successes

FCs and PCs mentioned that a decrease in apathy is often short-lived and ends when the external stimulation stops. However, they stated that when they were aware of and appreciated little successes (of meaningful interaction) this was rewarding for them. It motivated them to try different strategies and activities to interrupt apathy in a person with dementia. This was described as ‘*the effort that makes it worthwhile’*.

*‘I know she doesn’t want to do anything. So, what we’ll always do if we’re here, we go drink a cup of coffee downstairs or take a little walk. That is about all that is needed. We – my brothers and me – once took her to the zoo. We thought we would do her a favour, so we took her around but she wasn’t even looking at the animals. Instead, she said “Can we please go home?” (FC,001)*

*‘The moment we achieve something very small and I feel good about it and the resident does too, then this reflects on the resident. In contrast, if you achieve something small and you yourself don't feel it is good enough, that also has an effect on the resident.’ (PC,009)*

By sharing information and learning from each other, FCs and PCs stated that they were able to expand successful experiences in interrupting apathy.

*‘At a certain moment, we discovered that a resident spontaneously started knitting once we gave her knitting needles. So we took a picture and sent that to her family. They responded surprised: “Does our mom still know how to knit? Yes, your mother can still knit!“ It’s those little things we learned to enjoy more.’ (PC, P002)*

## Discussion

To the best of our knowledge, this study is the first to explore experiences of PwA. FCs and PCs regarding identifying and managing apathy in NHs. We found three themes that relate to the experiences of the stakeholders: (1) the challenge to appraise signals, (2) the perceived impact on well-being, (3) and applied strategies to manage apathy.

Regarding the first theme*,* our study confirms that all stakeholders relate to the description of the different domains of apathy by Miller et al. [52]. Nevertheless, we found that FCs as well as PCs have difficulties in identifying and appraising signals of apathy adequately in people with dementia. Different aspects seem to relate to this. For a start, although, PwA do recognize change within themselves, they cannot reflect on the consequences of apathy, nor express it actively. At the same time, FCs and PCs find it difficult to detect diminished or absent emotions and behaviour. However, if FCs and PCs for example know the character, life history and social preferences of the PwA, this helps them to recognize changes in the resident’s behaviour. This is important because caregivers do tend to realise the relevance of signals of apathy and (re)act upon them when they believe these signals represent a significant change [53, 54] or when they estimate apathy has a negative impact on the well-being of the PwA. So, the resident context is important when interpreting signals of apathy. Nevertheless, FCs and PCs may be uncertain when and to what extent the treatment of apathy is relevant. In line with recent literature, this is especially true in light off the needs and compelling behaviour of other residents with dementia that also require attention [22, 55] or when they see apathy as a natural phenomenon of (advanced) dementia [53, 56].

The second theme we found concerns the perceived impact of apathy on well-being. FCs and PCs think apathy has a negative effect on the well-being of a person with dementia when it reflects a decline or loss of abilities compared to the person’s previously more independent, socially engaged or active behaviour. In contrast to the study of Baber et al. [23], the PwA in our study did not express that apathy influenced their well-being. This matches known literature and underlines that apathy is usually reported as more impactful from the proxy perspective than from the perspective of the PwA [19, 57, 58]. Additionally, in line with other research comparing the burden of NPS [54], our study shows that PC do not express apathy as burdensome [26, 27, 54]. Nonetheless, both caregiver groups in our study describe that they experience frustration, disappointment, insecurity or withdrawal due to the lack of engagement with the PwA. This confirms previous findings that apathy negatively influences quality of life of FCs and PCs especially when they experience incompetence, insufficient skills and capabilities or negative feelings when supporting the persons with apathy [24, 18, 55, 59]. In FCs of home-dwelling people with apathy, avoiding or reducing deception or other negative feelings was even found to be a subconscious motivator to avoid the PwA [24]. For the FCs and PCs in our study, these negative feelings may make visiting or caring for a PwA difficult, as dealing with apathy requires effort and perseverance.

The third theme of our study shows that several caregivers have the skills and capabilities to apply specific strategies to manage apathy in a person with dementia. They do this by stimulating meaningful contact, adjusting expectation and appreciating little successes*.* However, for most FCs and PCs it is difficult to make or maintain a meaningful connection with the PwA when visiting or taking care, an experience that is shared with many FCs of people with dementia [60, 61]. Additionally, our study shows that FCs and PCs experience doubt as they want to offer the PwA a choice of whether or not to participate in activities or interaction, while they know from experience that PwA are unable to overcome apathy without external stimulation. This struggle is also experienced by spouses of community-dwelling people with apathy and dementia [24]. Our findings emphasize, in line with previous research, the importance of remaining engaged in meaningful activities and being involved in social interaction as important sources for the well-being of PwA [23, 62].

This study shows that PwA and dementia in NHs have difficulties in expressing their actual needs, starting goal directed behaviour and remaining involved in social interaction, which emphasizes their dependence on others for support and external stimulation to interrupt apathy. Systematic follow-up research on the long-term effects of treatment for apathy is lacking [63] but clinical experience suggests that although apathy can be momentarily interrupted, resolving it permanently may not be possible in daily care for people with dementia in NHs as the effect on apathy seems to wane unless activities or stimulation are continued. The absence of visitors and reduction of activities due to the restrictive measures during the COVID-19 pandemic (first wave), for example, led to an increase in apathy in nursing home residents [42]. Our findings suggest that in some residents with dementia apathy became more apparent as it was no longer interrupted by the external factors or reinforcing social interactions. Previous research showed that apathy can be interrupted when sufficient small-scale, individualized and person-centred activities are provided, social stimuli are well dosed and balanced and environmental factors are taken into account [8, 15, 43, 64]. Nevertheless, by focusing on what is possible in dealing with apathy instead of what is no longer possible, and by empowering FCs and PCs, people with dementia can be supported in maintaining their engagement in activities and social contact. Our findings indicate that educating FCs and PCs could increase the awareness and identification of apathy in NHs. Moreover, it seems important that FCs and PCs are supported to develop skills and capabilities to apply successful strategies to manage apathy in a person with dementia. The results of our study can thus direct the future development of psycho-social interventions for apathy.

### Strengths and limitations

One key strength of this study is the broad exploration of experiences with apathy and how FCs and PCs deal with it. Including participants from different professional backgrounds reduces the influence of preliminary education and can be helpful to determine how an intervention can best match training courses of different PCs as suggested in recent literature [65]. Another strength is the way in which this qualitative study was conducted, with data triangulation applied through the combination of interviews and focus groups which provided in broad and deep experiences from the participants. Experiences regarding apathy were explored within as well as between interviews and focus groups until saturation was achieved. Moreover, we used the deplorable yet unique situation of the restrictive measures in NHs due to COVID-19 to deepen the understanding of caregivers’ experiences with apathy.

However, some limitations must be mentioned. Unfortunately, we could only include a few PwA and FCs. For PwA, our criterion ‘to be able to communicate and reflect on their experiences with apathy’ limited inclusion. Due to issues with distance, mobility, health and COVID-19-restrictions, FCs were unable to participate in a focus group. This reduced representativeness of the results from the perspective of the PwA and their FCs and generalizations must be made with caution. Another potential limitation of this study is that the prevalence of apathy in participating NHs appeared lower than we expected based on previous studies. The PCs in this study reported difficulties in identifying apathy using the AES-10 in people with severe dementia. This may have played a role, and is in line with previous research highlighting the challenges in accurate apathy assessment in people with dementia in long-term care [58].

## Conclusions and implications

Based on the perspectives of PwA, FCs and PCs, we can conclude that all stakeholders are familiar with apathy as formulated in the diagnostic criteria for apathy in dementia, although oftentimes they do not know the term ‘apathy’. Appraising signals of apathy in people with dementia is challenging and this complicates the identification of apathy as significant NPS. However, it is important that apathy in people with dementia living in NHs is considered a relevant problem that needs attending to. FCs and PCs estimate that apathy negatively influences the well-being of the person with dementia, while PwA themselves only report a change to the person they were before. Moreover, apathy in a person with dementia has a negative impact on the well-being of both caregiver groups, as they experience negative feelings while dealing with apathy. The current study adds to the growing body of literature on apathy and how this relates to well-being, especially in FCs and PCs. FCs and PCs that have the skills and capabilities to apply specific strategies to manage apathy successfully can positively influence their own well-being when taking care or visiting a PwA. Our study shows that apathy—although briefly—can be interrupted successfully and repeatedly, when FCs and PCs apply strategies like stimulating meaningful contact, adjusting expectations and appreciating little successes. When interrupting apathy it is important that caregivers keep balance between under-stimulating (thereby maintaining apathy) and over-stimulating PwA, who -like all people- sometimes need moments to just do nothing. Future research is needed to support identification and appraisal of signals of apathy in people with dementia in long-term care and explore how FCs and PCs can be supported to positively interact and perform activities with a PwA. The results of this study provide a basis for developing a psycho-social intervention for FCs and PCs to identify and manage apathy in people with dementia in NHs.

### Supplementary Information

**Additional file 1. **Consolidated Criteria for Reporting Qualitative Research (COREQ): 32-item list

**Additional file 2. **Overview of themes, subthemes and additional illustrative quotes

**Additional file 3. **Topic lists on recognizing and dealing with apathy (interviews and focus group discussions). Topic lists on recognizing and dealing with apathy (Additional questions post Covid-19 interviews

## Data Availability

The dataset generated and/or analysed during this study are not publicly available in the interest of participant privacy and confidentiality but available from the corresponding author upon reasonable request.

## Abbreviations

PwA: Person with apathy
FC: Family Caregiver
PC: Professional caregiver
NH: Nursing Home
COVID-19: Coronavirus disease 2019
SABA: Shared Action for Breaking through Apathy
