# Supplementary material for: Identifying and managing apathy in people with dementia living in nursing homes: a qualitative study
Source: BMC Geriatr. 2023 Nov 9;23:727. doi: 10.1186/s12877-023-04422-y (PMC10636808; doi:10.1186/s12877-023-04422-y)
Supplement: Supplementary file 1 — Additional file 1. Consolidated Criteria for Reporting Qualitative Research (COREQ): 32-item list [file 12877_2023_4422_MOESM1_ESM.docx]

# Additional file 1 Consolidated Criteria for Reporting Qualitative Research (COREQ): 32-item list

|  | **Topic** | **Description** | **This study** | Section |
| --- | --- | --- | --- | --- |
|  | Domain 1: research team and reflexivity | | |  |
|  | *Personal characteristics* | | |  |
| 1. | Interviewer/facilitator | Which author/s conducted the interview or focus group? | Interviews HN; AP  Focus group Discussions HN, AP, AB | 5 |
| 2. | Credentials | What were the researcher’s credentials? *E.g. PhD, MD* | HN: PhD student;  MS Professor, MD, PhD;  AP MD, PhD;  RK, Professor, MD PhD;  RL, PhD; Associate Professor  DG Professor, PhD;  AB: PhD (moderator at 1 focus group) | Author checklist |
| 3. | Occupation | What was their occupation at the time of the study? | HN: licenced clinical neuropsychologist + PhD student  MS: MD, postdoc researcher  AP: postdoc researcher  RK: professor, postdoc researcher  DG: professor, postdoc researcher  AB: postdoc researcher |  |
| 4. | Gender | Was the researcher male or female? | HN,AP, DG are female, MS, RK, RL are male |  |
| 5. | Experience and training | What experience or training did the researcher have? | HN: Psychology, experienced and licensed clinical neuropsychologist, Followed additional Course ‘Training performing qualitative research with interviews and focus groups’ before the study  AP: Medicine, Elderly Care Physician (not practicing). Experienced post doc researcher in qualitative research.  Research team:  The researchers have diverse backgrounds; medical (RK, MS, AP) and psychological (DG, RL, HN) and are all specialized in elder care. All have a rich research experience in qualitative as well as quantitative research and clinical experience in nursing homes. | Author checklist |
|  | Relationship with participants | | |  |
| 6 | Relationship established | Was a relationship established prior to study commencement? | HN+ AP did not know the people with apathy or their family caregivers.  HN knew one professional caregiver before interviewing from the past as (former) colleague. In the last 5 years she did not collaborate with this participant. AP did not know any of the participants before the study. |  |
| 7 | Participant knowledge of the interviewer | What did the participant know about the researcher? *E.g. personal goals, reasons for doing the research* | Informed Consent Statement  At the start of the interviews participants were informed of the rationale of the research and were informed of HN being a PhD candidate. | methods |
| 8 | Interviewer characteristics | What characteristics were reported about the interviewer/facilitator*? E.g. bias, assumptions, research and interests in the research topic* | It was written in the invitational letter and orally stated that the researchers had no conflict of interest with the outcome of SABA.  The research team is particularly concerned about the low identification rate of apathy in nursing homes whilst apathy is very common and has severe outcomes |  |
|  | Domain 2: study design | | |  |
|  | *Theoretical framework* | | |  |
| 9 | Methodological orientation and theory | What methodological orientation was stated to underpin the study? *E.g. grounded theory, discourse analysis, ethnography, phenomenology, content analysis* | Generic Qualitative Research Approach (Kahle, 2014), using descriptive approach and inductive thematic analysis during the iterative process of data analysis | methods |
|  | *Participant selection* | | |  |
| 10 | Sampling | How were participants selected? *E.g. purposive, convenience, consecutive, snowball* | Purposive sampling   - By conducting purposive sampling, a sample of people with apathy and dementia, family caregivers and professional caregivers with different professions (nurses, psychologists, physicians, activities coordinator) with variation in age, sex, cultural background and educational level was obtained. Professions represent a cross-section of processionals in Dutch NHs involved in daily care for PwA | methods |
| 11 | Method of approach | How were participants approached? *E.g. face-to-face, telephone, mail, email* | Face-to-face and/or by telephone and/or e-mail   - Of the residents and their family caregivers who met the inclusion criteria, those who were able to participate in an interview and communicate about apathy were approached face-to-face and/or by telephone and/or e-mail for participation by a familiar nurse or psychologist. - The professional caregivers were approached face-to-face and/or by telephone and/or e-mail by the local coordinator at the participating organization. - The participants of the additional interviews after the first COVID-19 lockdown were approached by e-mail. They specifically gave permission to be approached for other research projects after they had voluntarily participated in a study on the impact of COVID-19 lockdown measurements on behavioral problems in Dutch nursing homes - All interviews and focus groups were conducted in Dutch. Translation of the quotes was done by a registered translation office and checked by an native speaker | methods |
| 12 | Sample size | How many participants were in the study? | 40 (of whom 7 participated more than ones, see question 18)   - 11 in-depth interviews (2 people with apathy and dementia,   3 family caregivers, 6 professional caregivers)   - 12 professional caregivers in 2 focus group discussions ( N=5 and N=6) - 17 additional in-depth interviews after COVID-19 lockdown ( 1 family caregiver and 16 professional caregivers) | See table 1 for detailed information |
| 13 | Non-participation | How many people refused to participate or dropped out? Reasons? | 4 people with apathy and their caregivers refused to participate,   - Reasons for not participating were no interest(N=2), deceased (N=1) unknown(N=1) - 2 PwA and 3 FC consented to be interviewed, none dropped out   None of the approached professional caregivers declined |  |
|  | *Setting* | | |  |
| 14 | Setting of data collection | Where was the data collected? *E.g. home, clinic, workplace* | At location in the participating nursing home units + online   - People with dementia and apathy and their family were interviewed together by the interviewer (HN) in their own room at the unit of residence. - The separate interviews and focus groups with professional caregivers took place at the participating nursing home. This was the workplace of the professional caregiver - Due to ongoing restrictions, the additional interviews after the first COVID-19 lockdown were held by HN and AP using digital connection (ZOOM, MSTeams) | methods |
| 15 | Presence of non-participants | Was anyone else present besides the participants and researchers? | No   - The people with apathy and dementia were interviewed simultaneously with their family caregiver (including the legal representative) who also participated in the study. - Besides researchers and participants no one else was present at the focus group discussions |  |
| 16 | Description of sample | What are the important characteristics of the sample? *E.g. demographic data, date* | See table 1 for details of demographic characteristics | See results and table 1 |
|  | *Data collection* | | |  |
| 17 | Interview guide | Were questions, prompts, guides provided by the authors? Was it pilot tested? | An interview topic guide was used. The interview guide was not pilot tested   - A semi-structured in-depth interview guide was used, and at the end of the interview it was checked whether or not all topics were discussed and/ or if there were specific additional topics that needed addressing according to the participant. - Interviewees did not want to do have a written member check, but there was a verbal summary and member check performed at the end of each interview. | See additional file 1 Topic guide for details |
| 18 | Repeat interviews | Were repeat interviews carried out? If yes, how many? | Yes   - 23 participants were interviewed once. - 10 professional caregivers participated in one of two focus group discussion - Three professional caregivers participated in an interview and one focus group. - Three professional caregivers participated in first and second interview (post covid). - One family caregiver participated in both interviews and one focus group | See Table 1 for participants’ details |
| 19 | Audio/visual recording | Did the research use audio or visual recording to collect the data? | Yes: Audio recordings were used to collect the data   - All interviews were audio-taped (by HN) - Thereafter these recordings were transcribed verbatim (all by a research assistant) using the same instructions. - A selection of the transcripts were checked against the tape recordings (HN, AP) | methods |
| 20 | Field notes | Were field notes made during and/or after the interview or focus group? | Yes   - During the interview the topic guide was used as a prompt. Afterward each interview fieldnotes and a summary were written. - A topic guide was used during focus groups discussions . Afterwards fieldnotes and a summary was written | additional file 1 Topic guide for details |
| 21 | Duration | What was the duration of the interviews or focus group? | - Interviews lasted between 53-107 minutes - Both focus group discussions lasted 120 minutes including a 30 minute break - The additional online interviews (post COVID-19 lockdown) lasted between 39-104 minutes |  |
| 22 | Data saturation | Was data saturation discussed? | Yes   - Findings and data saturation was discussed with the research group regularly ( DG, HN, AP, RK, MS, RL) . Data saturation was reached when no new ideas - Input from interviews was used in focus groups discussions to further explore, broaden and deepen the understanding of experiences of PCs | methods |
| 23 | Transcripts returned | Were transcripts returned to participants for comment and/or correction? | No:   - At the end of the interview a verbal summary was given and checked and it was offered to receive a transcript for comment and/or correction. None of the participants required a written transcript | methods |
|  | Domain 3: analysis and findings | | |  |
|  | *Data analysis* | | |  |
| 24 | Number of data coders | How many data coders coded the data? | Two   - Researchers (HN, AP) separately coded the data |  |
| 25 | Description of the coding tree | Did authors provide a description of the coding tree? | Yes   - See additional file 2 for an overview of themes, subthemes and codes | Results + additional file 2 |
| 26 | Derivation of themes | Were themes identified in advance or derived from the data? | Three themes were derived from the data. An overview of themes, categories and codes is provided in the article text and additional quotes are provided in additional file 2 | results |
| 27 | Software | What software, if applicable, was used to manage the data? | Analysis was done with Atlas.ti 8.4.20 (Atlas.ti Scientific Software Development, Berlin, Germany) | methods |
| 28 | Participant checking | Did participants provide feedback on the findings? | Yes   - At the end of each interview the participants a summary was given by the interviewer. Additionally al participants were asked if they would like to receive a transcript of the interview to provide feedback, which none of them wanted. - Feedback on the findings was given by the research team and the adversary expert team comprising two professional caregivers and one family caregiver | methods |
|  | *Reporting* | | |  |
| 29 | Quotations presented | Were participant quotations presented to illustrate the themes/findings? Was each quotation identified? *E.g. participant number* | Yes, see results section and Additional File 2 | results |
| 30 | Data and findings consistent | Was there consistency between the data presented and the findings? | Yes.   - In the results section quotations are used to illustrate the consistency | results, discussion and Additional File 2 |
| 31 | Clarity of major themes | Were major themes clearly presented in the findings? | Yes | results, discussion and Additional File 2 |
| 32 | Clarity of minor themes | Is there a description of diverse cases of discussion of minor themes? | Yes   - Whitin the selected data, diverse cases are described and minor themes discussed | results and discussion |

Developed from: Allison Tong, Peter Sainsbury, Jonathan Craig, Consolidated criteria for reporting qualitative research (COREQ): a 32-item checklist for interviews and focus groups, International Journal for Quality in Health Care, Volume 19, Issue 6, December 2007, Pages 349–357, <https://doi.org/10.1093/intqhc/mzm042>
