# Supplementary material for: Identifying and managing apathy in people with dementia living in nursing homes: a qualitative study
Source: BMC Geriatr. 2023 Nov 9;23:727. doi: 10.1186/s12877-023-04422-y (PMC10636808; doi:10.1186/s12877-023-04422-y)
Supplement: Supplementary file 2 — Additional file 2. Overview of themes, subthemes and additional illustrative quotes [file 12877_2023_4422_MOESM2_ESM.docx]

# Additional File 2. Overview of themes, subthemes and additional illustrative quotes

| Themes (heading) and subthemes | | | | | Illustrative quotes | | |  |  |  |
| --- | --- | --- | --- | --- | --- | --- | --- | --- | --- | --- |
| The challenge to appraise signals | | | | | | | | | |  |
|  | Perceiving loss of emotions and behaviour | | | | | *‘I’ve heard the term [apathy]. Actually, very briefly, being absent. (FC,003) And what do you mean by that? (I) Being a little nonchalant. Indifferent. (FC,003)*  *‘Often these are the people who do not stand out and who are no burden to other people.’ (PC,001)*  *‘How do I notice apathy? What I myself notice is that I’ll recognize apathy by the family's remarks. Because they know the person better and then they make comments like “She is so withdrawn, or she takes so little action, she sits in the armchair most of the time”.’ (PC,003)* | | | |  |
|  | The importance of knowing the context | | | | | *‘Once somebody [with apathy] is doing something, then he/she can stay occupied.’ (PC,011)*  *‘I walk to the hallway and sit again. I used to knit a lot, well, they don’t see me doing that anymore. (PwA,002)*  *‘I think it [apathy]is far more noticeable now[during lockdown] in a lot of resedents, just because what countered it before fell away. Take that lady for example, who was visited by her husband every day, we saw her sitting listless, while she usually sits with her husband and they sometimes participate in activities.‘ (PC,004)* | | | |  |
|  |  | | | | |  | | | |  |
|  | | Apathy as part of dementia | | | | *‘I also think the more people progress in their dementia, the more the question raises whether they are still aware of the things happening around them or are they more in their own world? But in my opinion that doesn't necessarily mean those people have apathy.’ (PC,006)*  *‘I think there are different kinds [of apathy]. Those that occur suddenly. And indeed, with depression someone becomes more apathetic or with advanced dementia someone [with apathy] turns more and more into his own world and becomes more closed off from the environment.’ (PC,007)* | | | |  |
| Perceived impact on wellbeing | | | | | | | | | |  |
|  | | Perceived impact of apathy on well-being of a PwA | | | | *‘Well sometimes you plod, you walk to the living room and then, I guess, they [staff] ask if you need something, but you won’t ask it for yourself. (FC,001) No, not by myself. (PwA,001).’*  *‘And if you find that it is difficult to start on your own? (I) I don’t experience that. (PwA,001) You don't find it hard that you aren’t able to start the day, don’t have lots of interest in things? (I) No, I always try, always did, by the way.’ (PwA,001)* | | | |  |
|  | |  | | | | *‘If someone really doesn't initiate anything at all and if that's a problem, when we see that a resident suffers from it, then we'll discuss it.’ (PC,015)*  *‘Well, if you don’t initiate anything at all, when you have no contact with others, when your world is so empty…What do you have? Then, you just sit there and that’s it. That, to me, really seems terrible.’ (PC,005)* | | | |  |
|  | | Perceived impact of apathy on the well-being of the FC and PC | | | | *‘At first this [decline in activities] was annoying for me, I thought “Mother come on!” But then I realised it’s not that she doesn’t want to, she just isn’t able anymore.‘ (FC,003)*  *I think for professional caregivers and family apathy oftentimes is challenging because you’ll put effort in it, and get nothing in return. Then, it becomes a [viscous] circle in which someone [with apathy] decreases, because you think “It doesn’t matter if I put energy in, the person won’t respond anyway, so I don’t need to anymore. And then the behaviour gets worse and worse. (PC, 016)* | | | |  |
| Applied strategies to manage apathy | | | | | | |  | | |  |
|  | | | Stimulating meaningful contact | | | *‘Not outside the unit, but inside the unit, you can achieve beautiful moments. When you can break through the apathy of [someone] who never wants anything, who always says: “leave me be” or “it’s not necessary”, and then one afternoon Spotify is on and you find yourself having the time of your life. And then you can say [to the PwA] “we really enjoyed ourselves, didn’t we?” (PC, 006)*  *‘I saw a resident with a stone-like expression and this person – of course – said he didn’t want to go anywere, doesn’t feel up to anything, doesn’t want to eat, wants to go to bed, and one morning I sat with him, just making small talk and then suddenly I see a smile on his face and then it’s all different. And that stays for half an hour until I leave and then its just the way it was before, before I sat down with him.’ (PC,010)* | | | | |
|  | | | Adjusting expectations | | | *‘I don’t think you can totally take it [apathy] away, because someone with severe dementia can’t be “on” all of the time, they cannot. I think when now and then, there are those moments of contact, pleasant activities and once in a while you see somebody is enjoying themselves, then I think you have come a long way.’ (PC,005)*  *‘I think sometimes we just expect too much in terms of interaction. That just being present is enough. Just sit with somebody, perhaps reading but you are there. Or you read the paper and once in a while you say something like “Oh look what it reads here...this and that.. it’s something right?” And then you move on. You are present and you feel the connection.’ (PC,012)* | | | | |
|  | | | Appreciating little successes | *We often think activities should be huge, or we must do a lot, but sometimes there is enough there already, like the smell of food can give a positive stimulus (PC,009)*  *‘And how can you gauge if something you do helps in case of apathy [in a person with dementia]? (I). By the little success you achieve, the little things. When you see somebody [with apathy] brighten up, sit up, still go to an activity and afterwards being cheerful and making small talk.’ (PC,006)* | | | | |  |  |

# Additional File 2. Overview of themes, subthemes and additional illustrative quotes

# Themes (bold), subthemes (underlined) and additional illustrative quotes (*italic*)

PwA = person with apathy and dementia; FC = family caregiver; PC = professional caregiver; I = interviewer
