# Supplementary material for: Identifying and managing apathy in people with dementia living in nursing homes: a qualitative study
Source: BMC Geriatr. 2023 Nov 9;23:727. doi: 10.1186/s12877-023-04422-y (PMC10636808; doi:10.1186/s12877-023-04422-y)
Supplement: Supplementary file 3 — Additional file 3. Topic lists on recognizing and dealing with apathy (interviews and focus group discussions). Topic lists on recognizing and dealing with apathy (Additional questions post Covid-19 interviews [file 12877_2023_4422_MOESM3_ESM.docx]

**Topic lists on recognizing and dealing with apathy (interviews and focus group discussions)**

**Person with apathy and dementia**

*Topic: recognizing apathy*

- What comes to your mind thinking of the term apathy?
- What causes apathy in your opinion?
- How do you notice apathy in yourself?
- How can others recognize apathy in you?

*Topic: Experiences in dealing with apathy:*

- How does apathy affect you?
- How do you deal with apathy in daily life?
- What supports you in dealing with apathy?
- Can you explain if it’s difficult/burdensome to deal with apathy or not?

**Family caregiver and Professional caregiver**

*Topic: Recognizing apathy*

- What comes to your mind thinking of the term apathy?
- What causes apathy in your opinion?
- How do you notice apathy in the person with dementia?
- How can professional/family caregivers recognize apathy in people with dementia?
- What was your response when you were told/ discovered the person with dementia had apathy?

*Topic: Experiences in dealing with apathy:*

- How does apathy affect you?
- How do you deal with apathy in daily life / in your work?
- What supports you – as a family/professional caregiver- in dealing with apathy?
- What do you need - as a family/professional caregiver - to take good care off a person with dementia and apathy?
- Can you explain if it’s difficult/burdensome to deal with apathy or not?

**Topic lists on recognizing and dealing with apathy (Additional questions post Covid-19 interviews**

**Family caregiver and Professional Caregivers**

*Topic: Recognizing apathy*

- To your opinion, how did the COVID-19 measures influence apathy in people with dementia?
- In what way has your own opinion changed, about apathy in people with dementia?
- Can you explain if and how your recognition of apathy has changed due to the COVID-19 lockdown?

*Topic: Experiences in dealing with apathy:*

- If so, how did the COVID-19 measures affect apathy in people with dementia?
- How did you experience dealing with apathy during this period?
- If so, how was apathy affected after the visitors-ban terminated?
